# Use of the McIsaac Score to Predict Group A Streptococcal Pharyngitis in Outpatient Nurse Phone Triage and Electronic Visits Compared With In-Person Visits: Retrospective Observational Study

**DOI:** 10.2196/25899

**Published:** 2021-12-20

**Authors:** Jennifer L Pecina, Leah M Nigon, Kristine S Penza, Martha A Murray, Beckie J Kronebusch, Nathaniel E Miller, Teresa B Jensen

**Affiliations:** 1 Department of Family Medicine Mayo Clinic Rochester, MN United States; 2 Department of Nursing Mayo Clinic Rochester, MN United States; 3 Mayo Clinic Express Care Mayo Clinic Rochester, MN United States

**Keywords:** strep pharygitis, e-visit, electronic visit, telemedicine, telecare, virtual visit, McIssac score, nurse phone triage, scoring system, sore throat, group A streptococcus, telehealth, nurse, phone, triage

## Abstract

**Background:**

The McIsaac criteria are a validated scoring system used to determine the likelihood of an acute sore throat being caused by group A streptococcus (GAS) to stratify patients who need strep testing.

**Objective:**

We aim to compare McIsaac criteria obtained during face-to-face (f2f) and non-f2f encounters.

**Methods:**

This retrospective study compared the percentage of positive GAS tests by McIsaac score for scores calculated during nurse protocol phone encounters, e-visits (electronic visits), and in person f2f clinic visits.

**Results:**

There was no difference in percentages of positive strep tests between encounter types for any of the McIsaac scores. There were significantly more phone and e-visit encounters with any missing score components compared with f2f visits. For individual score components, there were significantly fewer e-visits missing fever and cough information compared with phone encounters and f2f encounters. F2f encounters were significantly less likely to be missing descriptions of tonsils and lymphadenopathy compared with phone and e-visit encounters. McIsaac scores of 4 had positive GAS rates of 55% to 68% across encounter types. There were 4 encounters not missing any score components with a McIsaac score of 0. None of these 4 encounters had a positive GAS test.

**Conclusions:**

McIsaac scores of 4 collected during non-f2f care could be used to consider empiric treatment for GAS without testing if significant barriers to testing exist such as the COVID-19 pandemic or geographic barriers. Future studies should evaluate further whether non-f2f encounters with McIsaac scores of 0 can be safely excluded from GAS testing.

## Introduction

The McIsaac (modified Centor) scoring system is a validated tool used to determine the likelihood of an acute sore throat being from group A streptococcus (GAS) [1-3]. Criterion include patient age, absence of cough, fever, tonsillar swelling or exudates, and the presence of anterior cervical lymphadenopathy, with the last 2 criteria requiring a physical examination [1]. Even with 4 of the criteria present, the likelihood of an infection with strep is around 60% [4], so best practice dictates patients should be swabbed for GAS prior to initiating treatment with an antibiotic. Current guidelines also suggest against GAS testing in patients with a McIsaac score of less than 3 [5,6]. The most recent Infectious Diseases Society of America guidelines recommend considering testing unless other features strongly suggest a viral etiology [7].

The McIsaac criteria were based on clinician assessment and were not intended to be based on patient/caregiver report. The ability of patients and parents to assess tonsils and lymph nodes is not well studied. Minimal evidence exists in the literature related to use of the McIsaac criteria being calculated based on patient/caregiver report in a non–face-to-face (non-f2f) encounter. A previous study showed that adult patients may over- or underreport physical exam findings compared with clinicians [8]. Another study comparing patients or caregivers to physicians showed moderate to substantial agreement for 3 of 4 key pharyngitis signs and symptoms [9].

In 2017, our institution began using the McIsaac scoring criteria for non-f2f nurse phone triage and e-visit encounters. This scoring system was chosen due to it being validated and having relatively few components needing a response from patients/caregivers. Both phone triage and e-visit encounters relied on patients/caregivers to report on historical symptoms of cough and fever as well as physical exam findings of enlarged tonsils, tonsillar exudate, and anterior cervical lymphadenopathy. Both phone triage encounters and e-visits also allowed patients/caregivers to respond with “I don’t know” for any of the McIsaac criteria with the exception of e-visits not allowing an “I don’t know” response for cough. e-Visits were text-based asynchronous visits that did not require photo or video capability. To complete an e-visit, patients/caregivers completed an online questionnaire via a secure patient portal which was then reviewed by family medicine nurse practitioners and physician assistants. Both e-visit and phone triage end points recommended reporting for strep testing if the McIsaac score was 3 or higher. However, some patients were also referred for a f2f visit or strep testing with scores less than 3 either by clinician judgement or, at times, due to patient/caregiver desire. Additionally, triage nurses sent patients for strep testing if the patient or caregiver was not able to assess any components of the score.

The primary aim of this study was to evaluate and compare use of the McIsaac criteria to predict positive GAS tests in e-visits, phone triage, and f2f visits. We hypothesized that there would not be a significant difference in positive GAS results by McIsaac score between the different encounter types.

## Methods

This retrospective, observational study evaluated patients, ages 3 to 75 years, who had a nurse triage phone encounter, submitted an e-visit, or had a f2f visit for acute sore throat between February 23, 2017, and May 4, 2018, and had subsequent GAS testing done on the same day as the encounter. GAS testing was done using a polymerase chain reaction test. During the study time period, there were 211 e-visits with same day strep tests. All 211 e-visit encounters and a randomly selected sample of 211 records for both phone and f2f encounters were manually reviewed to determine the McIsaac score by patient/caregiver self-assessment during non-f2f encounters (nurse phone triage and e-visits) and by clinicians at f2f encounters. For f2f encounters, a score of 1 for fever was given if the provider included in their history that the patient reported a fever or the vital signs for the visit included a temperature greater than 38 °C. F2f visits without a fever at the time of the visit and with no mention in the note of the patient/caregiver reporting presence or absence of fever were counted as missing data for fever. Scores for each encounter type were compared with GAS results. We also calculated receiver operating characteristic (ROC) curves and area under the curve (AUC) for the McIsaac score for each encounter type. Patients were excluded if they had previous treatment for GAS within 30 days prior to the encounter, were currently on antibiotics for acute infection, or were younger than age 3 years or older than age 75 years. Statistical methods used are listed in the result tables. JMP PRO (version 14.1.0, SAS Institute Inc) was used to perform statistical analysis. This study was approved by the Mayo Clinic institutional review board.

## Results

There was no difference in average patient age or percentage of positive strep tests between encounter types (Table 1).

There was a significantly higher percentage of patients aged 15 to 44 years and a lower percentage of patients aged 45 years and older with e-visits compared to phone call and f2f encounters. There were significantly more phone and e-visit encounters with missing score components compared with f2f visits. For individual score components, there were significantly less e-visits missing fever and cough information compared with phone call and f2f encounters. F2f encounters were significantly less likely to be missing descriptions of tonsils and lymphadenopathy compared with phone and e-visit encounters.

Percentages of positive strep tests for each McIsaac score by encounter type are shown in Table 2. There were no significant differences in percentages of positive strep tests between encounter types for any of the McIsaac scores.

We also reviewed encounters that had all components to document a McIsaac score (ie, no missing criteria). There were 342 encounters with no missing score elements (101 phone encounters, 83 e-visits and 158 f2f visits). Of these encounters, 52.1% (178/342) had a positive strep test. There were no significant differences between encounter types for percentage positive strep tests for all McIsaac scores (Table 3).

ROC AUC for e-visits was 0.62, for f2f was 0.69, and for phone encounters was 0.62.

**Table 1:** Patient age, positive strep tests, and missing score elements for all encounter types.

		Total (n=633)		Phone call (n=211)		e-Visit (n=211)		Face-to-face visit (n=211)		*P* value
Positive strep tests, n (%)		312 (49)		96 (45)		113 (53)		103 (49)		.25^a^
**Age (years), mean**		22.2		22		22.6		21.9		.86^b^
	3-14, n (%)	288 (45)	97 (46)		92 (44)		99 (47)		.78^a^	
	15-44, n (%)	294 (46)	91 (43)		113 (53)		90 (43)		.04^a^	
	≥45, n (%)	51 (8)	23 (11)		6 (3)		22 (10)		.001^a^	
**Missing McIsaac criteria, n (%)**		291 (46)		110 (52)		128 (61)		53 (25)		<.001^a^
	Fever description	30 (5)	5 (17)		4 (2)		21 (10)		<.001^a^	
	Cough description	80 (13)	45 (21)		0		35 (17)		<.001^a^	
	Tonsil description	171 (27)	72 (34)		98 (46)		1 (1)		<.001^a^	
	Lymph node description	116 (18)	56 (26)		58 (28)		2 (1)		<.001^a^	

^a^Chi-square test.

^b^Analysis of variance.

**Table 2:** Percentage positive strep tests by encounter type and McIsaac score for all encounters.

McIsaac score	All, % (95% CI) [n pos/n total]	Phone call, % (95% CI) [n pos/n total]	e-Visit, % (95% CI) [n pos/n total]	Face to face, % (95% CI) [n pos/n total]	*P* value^a^
0	17 (7-37) [4/23]	21 (8-48) [3/14]	0 [0/2]	14 (3-51) [1/7)	.62
1	30 (20-42) [20/66]	35 (19-55) [8/23]	33 (14-61) [4/12]	26 (14-43) [8/31]	.75
2	35 (27-44) [44/125]	31 (18-47) [11/36]	42 (28-58) [17/40}	33 (21-47) [16/49]	.50
3	54 (47-60) [119/222]	53 (42-64) [40/76]	52 (42-62) [46/88]	57 (44-69) [33/58]	.85
4	63 (56-70) [125/197]	55 (42-67) [34/62]	67 (55-77) [46/69]	68 (56-78) [45/66]	.24
s2	31.8 (25.9-38.2) [68/214]	31.8 (25.9-38/3) [68/214]	38.9 (27-52.2) [21/54]	28.7 (20.3-39) [25/87]	.43

^a^Chi-square test.

**Table 3:** Percentage positive strep tests by encounter type and McIsaac score for 342 encounters with no missing McIsaac criteria.

McIsaac score	All, % (95% CI) [n pos/n total]	Phone call, % (95% CI) [n pos/n total]	e-Visit, % (95% CI) [n pos/n total]	Face to face, % (95% CI) [n pos/n total]	*P* value
0	0 [0/4]	0 [0/1]	—^a^	0 [0/3]	>.99^b^
1	27 (13.7-46.1) [7/26]	50 (15-85) [2/4]	—	22.7 10-43.4) [5/22]	.28^c^
2	28.3 (17.3-42.5) [13/46]	28.6 (8.2-64.1) [2/7]	22 (6.3-54.7) [2/9}	30 (16.6-47.9) [9/30}	.90^c^
3	49.1 (40-58.4) [53/108]	48.8 (34.2-63.5) [20/41]	40 (23.4-59.3) [10/25]	54.8 (40-68.8) [23/42]	.61^c^
4	66.5 (58.8-73.3) [105/158]	58.3 (44.3-71.1) [28/48]	71 (57.6-82.1) [35/49]	68.9 (56.4-79.1) [42/61]	.35^c^
≤2	26.3 (17.7-37.1) [20/76]	33.3 (13.8-60.9) [4/12]	22.2 (6.3-54.8) [2/9]	25.4 (15.8-38.3) [14/55]	.84^c^

^a^Not applicable.

^b^Fisher exact test.

^c^Chi-square test.

## Discussion

### Principal Findings

The use of nursing protocols and e-visits in today’s outpatient settings has improved access for patients and proven to be a patient satisfier [10-14]. Patients today want health care that fits their schedules with tests and treatments accomplished with minimal waiting [15,16]. The ability to access a triage nurse 24/7 or complete an e-visit with a response within a few hours has improved the care of outpatients and allowed more access for patients who need to be seen by a provider [10,13-15,17]. In addition, the cost of such care is greatly reduced from the usual clinic visit [11,13,14,18,19].

The validation of non-f2f visits for sore throats is important when determining the safety of this type of care. Potential ramifications of not treating GAS infections include the possibility of rheumatic fever leading to rheumatic heart disease, invasive GAS diseases, and acute glomerulonephritis [5,20-22]. GAS tonsillitis can also lead to significant discomfort [23]. Knowledge that a sore throat is due to a strep infection versus a viral cause could help reduce complications and get patients back to work or school after antibiotic treatment or home care for an upper respiratory viral infection.

Our study showed no significant difference in the percentage of positive strep tests for each McIsaac score when compared across f2f and non-f2f encounter types. Additionally, ROC AUC values were very similar for the McIsaac score across encounter types. These findings support the use of McIsaac criteria in non-f2f encounters as being comparable to use in f2f encounters. However, our study showed a high rate of positive strep tests for low McIsaac scores in all encounter types (including f2f visits) compared with the literature. Specifically, our rates of 0% to 21% positive strep tests for McIsaac scores of 0 are higher than the published values of 1% to 2.5% [1,4,24]. Similarly, for McIsaac scores of 1 our findings of a positive strep test of 26% to 35% across encounter types appears higher than the published literature of 5% to 10% [1,4,24]. This could be a result of our methodology of including patients who did not have complete data to calculate a McIsaac score and choosing to count missing data (either due to not being recorded in encounters or being answered as “I don’t know” in non-f2f encounters) as contributing 0 points to the total McIsaac score. We attempted to correct for this limitation by further analyzing only encounters with no missing data to compute a score. There were only 4 encounter types with all score components with McIsaac scores of 0, and none of these encounters had a positive strep test. There were only 26 encounters with no missing data and McIsaac scores of 1 with a positive strep rate of 22% to 50%. Another consideration for the higher percentage of positive strep tests is the presence of a carrier state since as many as 12% to 32% of children may be pharyngeal carriers of GAS [25,26]. A throat swab would be unable to differentiate whether the pharyngitis is due to acute GAS infection or viral illness in a GAS carrier [25-27].

In contrast to the above findings, the percentage of positive strep tests for McIsaac scores of 4 appears comparable to rates listed in the published literature for all encounter types (both for those not missing any score components and those with missing score components). Some previous guidelines have recommended consideration of empiric treatment for strep throat for patients with McIsaac scores of 4 or higher [24]. Our study lends support to empiric treatment of patients with McIsaac scores of 4 in non-f2f encounters, as the percentage of positive strep tests is high in this group without any significant differences between encounter types and is consistent with the published literature. This finding may be helpful in scenarios where f2f visits are challenging due to geographical barriers, reduced access to appointments in the clinics during influenza season, or even care barriers such as our current COVID-19 pandemic. Provider visits were more complete in documenting the McIsaac criteria than the phone triage or e-visits. In particular, presence or absence of tonsillar enlargement/exudate and cervical lymphadenopathy were more consistently noted in f2f visits then in non-f2f visits. However, e-visits were significantly less likely to be missing information on fever. No e-visits were missing data on the presence or absence of cough; however, this was due to the e-visit process not allowing an answer of “I don’t know” for cough. The e-visit consists of a series of questions to which the patient can answer yes, no, or “I don’t know” (for most answers), making the criteria easier to document. F2f clinic visits were not as likely as e-visits to document fever and cough. The clinic visits do not have a template for the McIsaac criteria, which most likely explains this difference in documentation.

There were no differences between encounter types in the average age of patients evaluated. Interestingly, however, patients aged 15 to 44 years more often used e-visits as their method of seeking care for a sore throat. This age group consistently uses the digital platform for health care at our institution more often than any other group. This reliance on electronic portals for health care has been the newest method to improve clinic access, reduce waiting time for patients seeking answers to health questions, and improve time spent on renewing prescriptions and allowing patients to access their health records anytime of the day or night. As this younger group ages, more electronic access to health care will need to be available.

### Limitations

Prima facie, our study showed a surprisingly high percentage of positive strep tests in the lower McIsaac scores as well as an overall high rate of positive GAS tests (49%). The high percentage of positive GAS tests in our study (both for low McIsaac scores and overall) is very likely due to the retrospective methodology used. In prospective studies of the McIsaac criteria, all patients have GAS testing done regardless of their McIsaac score or other risk factors for a positive GAS test [4,28]. Due to the retrospective nature of this study, only patients who had GAS testing recommended and done as part of their clinical care were included. This methodology means there are likely many patients with low McIsaac scores who were not represented in our dataset as they would have been had this been a prospective trial. Not including these patients with low McIsaac scores in our study (as they did not have GAS testing done) likely elevated the positive rate for the low McIsaac scores and thus the overall positive rate of tests as, in a prospective trial, these patients would have had GAS tests that would be more likely to have been negative. Specifically, we anticipate that patients with low McIsaac scores who were included in our study are likely to have had a higher pretest probability of having a positive GAS test compared with patients with low scores who were not recommended for testing. This higher pretest probability is likely since these patients were recommended to have GAS testing done despite a low McIsaac score. Reasons other than a high McIsaac score that could increase the pretest probability of having a positive test (and thus leading to these patients being tested) could include a history of positive GAS tests (either due to recurrent strep infections or carrier status) or a known strep contact. These factors would not be reflected in the McIsaac score but would lead to a higher pretest probability of testing positive for GAS and may have led to recommendations for these low McIsaac score patients to be tested, thus increasing the percentage of low McIsaac score patients with a positive GAS test. This would, in turn, elevate the overall positive GAS rate in our study. This supposition is further supported by the fact that our study has a lower percentage of patients with McIsaac scores of 0 and 1 (14% of our study population) when compared with a prospective multisite nationwide McIsaac validating study by Fine et al [4], which had 41% of their study population in the McIsaac 0 and 1 categories. A better comparison to validated studies would be comparing higher McIsaac scores of 3 or 4 where strep testing is routinely recommended. Our study found positive GAS rates of 54% and 63%, respectively, for these scores. This is closer to, although still somewhat higher than, the prospective study by Fine et al [4], where rates were 38% and 57%, respectively. Although somewhat higher, our study also included a significantly younger population (average 22.2 years) versus 34 years of age for Fine et al [4]. Given that younger patients have a higher pretest probability of a positive GAS test, this partially helps to explain the remaining discrepancy in the positivity rate [29].

### Conclusions

Measures to combat the above limitations could be a prospective trial performing GAS testing in all patients (including all patients with low McIsaac scores). Methods to improve non-f2f assessment of physical exam findings could include options for patients to send in photos of their tonsils for the e-visit and phone encounters for a provider to review and provide that component of the score. Additionally, photos with examples of various degrees of tonsillar hypertrophy and exudate could be included for patients to choose which appeared similar to their own exam findings. Descriptions (and diagrams with location) of cervical lymph node enlargement could also be included in e-visits to improve patient and caregiver assessment of this score component. Both changes have in fact been instituted in new processes at our institution.

There is no difference in the percentage of positive GAS tests for each McIsaac score when comparing f2f and non-f2f care. Our study is supportive that patients with a McIsaac score of 0 and no missing score components might be candidates to safely exclude from strep testing as none of these patients in our study had a positive strep test; however, as our numbers for this category are small this should be further evaluated in future studies. Additionally, McIsaac scores of 4 or higher calculated during non-f2f care could be considered for empiric treatment of GAS pharyngitis without confirmatory testing, especially when there are significant barriers to obtaining testing.

## Abbreviations

AUC: area under the curve
f2f: face-to-face
GAS: group A streptococcus
ROC: receiver operating characteristic
